# Comparative phylogenomics of *Streptococcus pneumoniae* isolated from invasive disease and nasopharyngeal carriage from West Africans

**DOI:** 10.1186/1471-2164-13-569

**Published:** 2012-10-29

**Authors:** Eric S Donkor, Richard A Stabler, Jason Hinds, Richard A Adegbola, Martin Antonio, Brendan W Wren

**Affiliations:** 1 Department of Pathogen Molecular Biology, London School of Hygiene and Tropical Medicine, London WC1E 7HT, UK; 2 Vaccinology Theme, Medical Research Council Unit, The Gambia; 3 Bacterial Microarray Group, St. George’s University of London, London, SW17 0RE, UK; 4 GlaxoSmithKline Vaccines, Wavre, Belgium; 5 Department of Microbiology, University of Ghana Medical School, Accra, Ghana

## Abstract

**Background:**

We applied comparative phylogenomics (whole genome comparisons of microbes using DNA microarrays combined with Bayesian-based phylogenies) to investigate *S. pneumoniae* isolates from West Africa, with the aim of providing insights into the pathogenicity and other features related to the biology of the organism. The strains investigated comprised a well defined collection of 58 invasive and carriage isolates that were sequenced typed and included eight different *S. pneumoniae* serotypes (1, 3, 5, 6A, 11, 14, 19 F and 23 F) of varying invasive disease potential.

**Results:**

The core genome of the isolates was estimated to be 38% and was mainly represented by gene functional categories associated with housekeeping functions. Comparison of the gene content of invasive and carriage isolates identified at least eleven potential genes that may be important in virulence including surface proteins, transport proteins, transcription factors and hypothetical proteins. Thirteen accessory regions (ARs) were also identified and did not show any loci association with the eleven virulence genes. Intraclonal diversity (isolates of the same serotype and MLST but expressing different patterns of ARs) was observed among some clones including ST 1233 (serotype 5), ST 3404 (serotype 5) and ST 3321 (serotype 14). A constructed phylogenetic tree of the isolates showed a high level of heterogeneity consistent with the frequent *S. pneumoniae* recombination. Despite this, a homogeneous clustering of all the serotype 1 strains was observed.

**Conclusions:**

Comparative phylogenomics of invasive and carriage *S. pneumoniae* isolates identified a number of putative virulence determinants that may be important in the progression of *S. pneumoniae* from the carriage phase to invasive disease. Virulence determinants that contribute to *S. pneumoniae* pathogenicity are likely to be distributed randomly throughout its genome rather than being clustered in dedicated loci or islands. Compared to other *S. pneumoniae* serotypes, serotype 1 appears most genetically uniform.

## Background

*Streptococcus pneumoniae* is part of the normal bacterial flora of the upper respiratory tract, but is also associated with severe invasive diseases, including meningitis, pneumonia and septicaemia as well as non-invasive diseases such as otitis media [1]. Transmission of *S. pneumoniae* occurs through respiratory droplets and is more commonly associated with healthy individuals who carry the organism in the upper respiratory tract [2,3]. Worldwide, the annual incidence of invasive pneumococcal disease (IPD) is about one million and though a global problem, the public health impact of IPD is higher in the developing world, where children less than 5 years of age are most affected [4,5].

The capsule is considered the main virulence determinant of *S. pneumoniae*, and only a few capsular types tend to be associated with invasive disease which is partly due to differential ability of the variant capsular types to resist phagocytosis [6,7]. Epidemiological evidence indicates that while some capsular types are often associated with invasive disease, some may be associated with carriage, while others are associated with both invasive disease and carriage [8-12]. In addition to the capsule, it is known that other pathogenic factors are required by *S. pneumoniae* for virulence [13], but the genetic factors that explain the pathogenesis and virulence of the organism is not fully understood.

Comparative whole genome analysis using DNA microarrays has been utilised to investigate several bacterial pathogens. The approach involves assessing the absence or presence of genes from strains based on reference genome(s) fixed to microarray, followed by robust statistical algorithms to infer the evolutionary relationships between test strains that is usually represented as a phylogenetic tree [14-18]. This allows interrogation of the genome content of bacterial strains from a variety of sources, environments and disease states, and the identification of genetic markers that may explain how different strains are adapted to their respective niches or disease capability. Few comparative genomics studies have been carried out on *S. pneumoniae*, and these studies were based mainly on strains from developed countries and none from Sub-Saharan Africa [19-23], where the organism exacts its greatest toll. Though these studies have contributed significantly to our understanding of *S. pneumoniae*, several aspects of the organism particularly, its pathogenicity, evolution and population structure in the Sub-Saharan Africa is still inadequately understood. In view of this, we carried out comparative phylogenomics (whole genome comparisons of microbes using DNA microarrays combined with Bayesian-based phylogenies) of 58 *S. pneumoniae* epidemiologically well defined isolates from West Africa with the aim of providing insights into the pathogenicity and other features related to the biology of the organism.

## Results and discussion

### Strain selection

**Figure 1:**
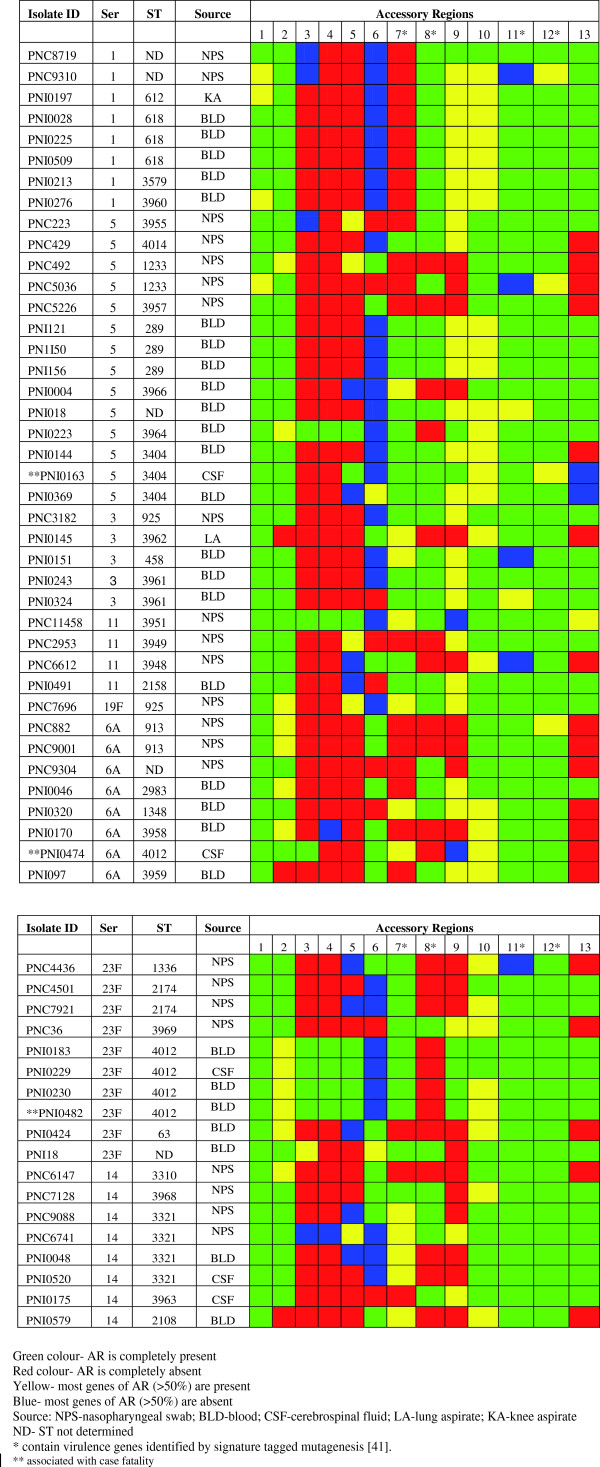
**Distribution of accessory regions among *S. pneumoniae *isolates of different serotypes and sequence types.**

A total of 58 isolates were used in this study, and were collected from three West African countries including The Gambia (52), Nigeria (4) and Ghana (2). All isolates were serotyped [24] and multilocus sequence typed [25] (Figure 1). The isolates comprised 35 invasive and 23 carriage isolates and were recovered from subjects of an age range of 3 months to 58 years. The carriage isolates were recovered from the nasopharynx of healthy human populations [9,26], while invasive isolates were recovered from specimens of blood (87%), CSF (10%) and lung and knee aspirates (3%) of patients with IPD [8,27,28]. Based on information from capsule serotype, the isolates were selected to cover pneumococcal serotypes of varying invasive disease potential in West Africa [8,9,26-29]. Eight serotypes were selected and included serotypes 1, 3, 5, 6A, 11, 14, 19 F and 23 F (Table 1). In West Africa, Serotypes 1 and 5 are common in IPD but rare in carriage and represent serotypes of high invasive disease potential; serotypes 3, 11 and 19 F are common in carriage but not in invasive disease, and represent serotypes of low invasive disease potential; serotypes 6A, 14, and 23 F are common in both invasive disease and carriage, and represent serotypes of intermediate disease potential. Overall, the isolates studied covered 35 different sequence types; invasive isolates covered 22 sequence types while the carriage isolates covered 16 sequence types.

**Table 1:** **Serotype distribution of invasive and carriage *S. pneumoniae *isolates used for comparative phylogenomics analysis**

**Serotype**	**No. of invasive isolates**	**No. of carriage isolates**	**Total**
1	6	2	8
3	4	1	5
5	9	5	14
6A	5	3	8
11	1	3	4
14	4	4	8
19 F	0	1	1
23 F	6	4	10
**Total**	**35**	**23**	**58**

### Core gene set of *S. pneumoniae* strains

Whole genome microarray comparisons of 58 isolates of *S. pneumoniae* were used to compute the minimal core gene set. This was achieved by calculating the total number of coding sequences (CDSs) that had a GACK (Genome Analysis by Charlie Kim) score of ‘present’ in every isolate and the control strain (TIGR4) using the advanced filtering function available in Genespring 6.1. The minimal core gene set for the *S. pneumoniae* isolates was 831 CDSs, which translates to 38% of the total genome of the isolates. Similarly, individual core gene sets were computed for invasive and carriage isolates and were found to be 1162 CDSs (84%) and 919 CDSs (63%) respectively (p < 0.05). The low core genome estimate of 38% observed in this study is quite similar to a core genome of 46% reported by Hiller *et al.*[22] but contrast significantly with a core genome of 73% reported by Obert *et al.*[21] and 80% reported by Tettelin *et al.*[20]. However, Hiller *et al.*[22] demonstrated that individual strains core orthologous clusters account for 68–79% of the genome. Reported core gene sets of some other streptococci species include 58% for *S. thermophilus*[30], 82.5% for *S. uberis*[31] and 82% for *S. agalactiae*[32]. Relatively low core gene of 28% has been reported for some non-streptococcal organisms such as *Yersinia enterocolitica*[16]. Thus core genome quantification may vary significantly among different bacterial strain collections and is highly dependent on the cut off method used as well as the core genome definition. The relatively low core genome reported in this study may reflect the more stringent approach used to compute the core genome (Section “Microarray data analysis and comparative phylogenomics”).

As expected, the *S. pneumoniae* core gene set was represented by many of the functional categories that are involved in housekeeping functions such as DNA metabolism, intermediary metabolism, protein synthesis and cellular processes. This concurs with other *S. pneumoniae* microarray studies [20-22]. Conserved housekeeping genes including those identified in the core genome of the West African isolates have been shown to be abundant in sequenced pneumococcal genomes [33-37]. Comparison of eight nasopharyngeal *S. pneumoniae* genomes with nine published genomes (including TIGR4 and R6) identified 1,454/3,170 (46% ) orthologous gene clusters conserved among all 17 strains [22]. The core genes consisted mainly of housekeeping genes but also contained 462 hypothetical proteins with no known function [22]. More than 70% of the West African *S. pneumoniae* core genes were present in the core gene set of Hiller *et al.*[22]. Virulence determinant CDSs including transport proteins and various enzymes such as hyaluronidase, neuraminidase A, phosphoglucomutase and triosephosphate isomerase were identified in the *S. pneumoniae* core gene set in this study. By comparison, hyaluronidase and neuraminidase A were also demonstrated to be conserved within the 17 genomes analysed by Hiller *et al.*[22]. The presence of virulence determinants in all the invasive as well as carriage isolates in the current study probably indicates that these virulence determinants are necessary, but not adequate, to determine the ability of an isolate to cause invasive disease. Also, analysis of the core gene set of the isolates showed that a wide range of mobile and extrachromosomal elements were conserved, which agrees generally, with information obtained from pneumococcal genomes that have been fully sequenced [20,33-37]. Within the Hiller *et al.* core gene list are twelve transposases listed [22], which were also present in the core gene list of our study.

### Putative virulence determinants and accessory regions

Overall, comparison of the gene content of invasive and carriage isolates identified at least eleven CDSs that were significantly associated with invasive isolates compared to carriage isolates (Table 2). IgA protease showed the largest difference between invasive and carriage isolates. This surface protein degrades IgA and thus helps *S. pneumoniae* to evade host immune system and provide an opportunity for more effective invasion [38,39]. Several transport proteins of the ABC type, were also significantly associated with invasive isolates and may be based on the fact that these transport proteins are involved in the transport of metal ions or nutrients which are required by pathogenic bacteria for growth and metabolic activities [40]**.** Several transcriptional genes were associated with invasive isolates/disease which has been previously reported [41]. Several other proteins were also associated with invasive isolates, but were mainly hypothetical proteins and therefore require further investigation.

**Table 2:** Genes that showed significant differences between invasive and carriage isolates

**Gene**	**Function**	**Frequency**	
		**Invasive**	**Carriage**
		**(N = 35)**	**(N = 23)**
SP0071	immunoglobulin A1 protease	35	2
SP0091	ABC transporter, permease protein	18	5
SP0161	hypothetical protein	25	7
SP0238	hypothetical protein	25	6
SP0491	hypothetical protein	26	9
SP0514	hypothetical protein	25	9
SP0743	transcriptional regulator	30	14
SP0955	competence protein	27	9
SP1032	iron-compound ABC transporter	25	5
SP1612	hypothetical protein	30	12
SP1800	transcriptional activator	31	14

N indicates total number of invasive or carriage isolates.

An accessory region was defined as three or more contiguous genes not conserved in all the isolates. Thirteen accessory regions (ARs) were identified in this study (Table 3) and the distribution of such regions among the study isolates is presented in Figure 1. By comparison previous studies have reported 13–38 ARs [21,42,43]. Nine ARs identified in the current study have been previously reported and include AR2, AR3, AR4, AR5, AR6, AR7, AR8, AR9 and AR13 [21,42,43]. Four ARs including AR1, AR10, AR11 and AR12 identified in this study have not been previously reported and represent novel ARs. In the case of AR1 and AR10, none of the genes in these regions have been associated with virulence and thus their functional role in virulence is not clear. Two of the novel ARs namely, AR11 and AR12 contained genes identified by Signature Tagged Mutagenesis (STM) as required for virulence in mice, but none of these ARs was associated with invasive disease [41]. The poor correlation of invasive isolates or serotypes of high invasive disease potential with ARs that contain virulence genes has also been reported by Bloomberg *et al.*[42]. In the study of Bloomberg *et al.*[42], though 24 ARs containing virulence genes were identified, only two of such ARs were preferentially found in invasive isolates or serotypes of high invasive disease potential.

**Table 3:** **Accessory regions identified among *S. pneumoniae *isolates**

**Accessory Region**	**TIGR4 locus**	**Gene annotation**
AR1	SP0482	hypothetical protein
	SP0483	ABC transporter, ATP-binding protein
	SP0484	hypothetical protein
AR2	SP1050	transcriptional regulator, putative
	SP1051	hypothetical protein
	SP1052	phosphoesterase, putative
AR3	SP1062	ABC transporter, ATP-binding protein
	SP1063	ABC-2 transporter, permease protein, putative
	SP1064	transposase, IS200 family
AR4	SP1129	integrase/recombinase, phage integrase family
	SP1130	transcriptional regulator
	SP1131	transcriptional regulator, putative
AR5	SP1134	hypothetical protein
	SP1135	hypothetical protein
	SP1136	hypothetical protein
	SP1137	GTP-binding protein, putative
AR6	SP1315	V-type ATP synthase subunit D
	SP1316	V-type ATP synthase subunit B
	SP1317	V-type ATP synthase subunit A
	SP1318	V-type ATP synthase subunit F
	SP1319	V-type sodium ATP synthase, subunit C
	SP1320	V-type sodium ATP synthase, subunit E
	SP1321	V-type ATP synthase subunit K
	SP1322	V-type ATP synthase subunit I
	SP1323	hypothetical protein
	SP1324	ROK family protein
	SP1325	oxidoreductase, Gfo/Idh/MocA family
	SP1326	neuraminidase, putative
	SP1327	hypothetical protein
	SP1328	sodium: solute symporter family protein
	SP1329	N-acetylneuraminate lyase
	SP1330	N-acetylmannosamine-6-phosphate 2-epimerase
	SP1331	phosphosugar-binding transcriptional regulator, putative
AR7	SP1341	ABC transporter, ATP-binding protein
	SP1342	toxin secretion ABC transporter, ATP-binding/permease protein
	SP1343*	prolyl oligopeptidase family protein
	SP1344*	hypothetical protein
AR8	SP1433	transcriptional regulator, AraC family
	SP1434*	ABC transporter, ATP-binding/permease protein
	SP1435	ABC transporter, ATP-binding protein
	SP1436	hypothetical protein
AR8	SP1437	hypothetical protein
	SP1438	ABC transporter, ATP-binding protein
AR9	SP1616	allulose-6-phosphate 3-epimerase
	SP1617	PTS system, IIC component
	SP1618	PTS system, IIB component
	SP1619	PTS system, IIA component
	SP1620	PTS system, nitrogen regulatory component IIA
	SP1621	putative transcription anti terminator BglG family protein
	SP1622	transposase, IS200 family
AR10	SP1738	guanylate kinase
	SP1739	hypothetical protein
	SP1740	hypothetical protein
AR11	SP1856*	transcriptional regulator, MerR family
	SP1857	cation efflux system protein
	SP1858	transcriptional regulator, TetR family
	SP1859*	transporter, putative
	SP1860	choline transporter
AR12	SP1896*	sugar ABC transporter, permease protein
	SP1897	sugar ABC transporter, sugar-binding protein
	SP1898*	alpha-galactosidase
AR13	SP2159	fucolectin-related protein
	SP2160	hypothetical protein
	SP2161	PTS system, IID component
	SP2162	PTS system, IIC component
	SP2163	PTS system, IIB component

* contain virulence genes identified by signature tagged mutagenesis [41].

Despite the poor correlation between invasive disease and ARs that contain virulence genes, differences in virulence between different clones of the same serotype could be explained by the distribution of such ARs in some cases (evidence provided below). This indicates that the role of ARs in pneumococcal virulence may be serotype dependent which has also been reported [21,42]. Included in this study, were four invasive isolates of the serotype 5 virulent PMEN clone ST 289, and also two serotype 5 carriage isolates of ST 1233 which is considered less virulent. The pattern of AR distribution of the ST 289 isolates was the same and carried all the ARs associated with virulence in this study (ARs 7, 8, 11 and 12). However, the ST 1233 isolates were deficient in three of the four ARs associated with virulence including AR7, AR8 and AR11. Thus these differences in ARs of the two serotype 5 clones may explain the enhanced virulence of ST 289. A similar observation has been reported for two serotype 19 F clones namely, ST 162 which is a virulent clone and ST 425, a non-virulent clone [42]. These observations also highlight the variations in virulence of clones of the same serotype and are important in pneumococcal vaccination, where virulent clones of a serotype rather than non-virulent clones of that serotype, undergo capsular switching and emerge with non-vaccine serotypes [44,45]. For an invasive serotype like serotype 5, it also shows that the ability of an isolate to cause invasive disease is not only dependent on the capsule type but also the genetic background of the strain.

Though ARs may have some relevance in pathogenicity, the extent to which ARs contribute to pneumococcal virulence is still not very clear. In this study, the 13 ARs identified did not show loci association with any of the eleven potential virulence genes identified. Analysis of the distribution of virulence genes identified by Hava and Camilli in TIGR4 indicates that the virulence genes did not cluster [41]. These observations suggest that virulence determinants that contribute to *S. pneumoniae* pathogenicity are likely to be distributed randomly throughout its genome rather than being clustered in dedicated loci or islands. This agrees with the findings of Obert *et al.*[21] which showed that ARs are more likely to adapt *S. pneumoniae* to carriage rather than invasive disease. Thus ARs may not play a highly prominent in pathogenicity as observed in pathogens such as uropathogenic *Escherichia coli*[46].

From Figure 1, it can be observed that some isolates of the same serotype and ST were found to express different patterns of ARs, which can be seen for ST 1233 (serotype 5), ST 3404 (serotype 5) and ST 3321 (serotype 14). This phenomenon of intraclonal diversity has also been observed in studies carried out by Silva *et al.*[43] and Bloomberg *et al.*[42], and shows that strains of the same serotype and ST may exhibit genetic and phenotypic differences. In the study by Silva *et al.*[43] different patterns of ARs was observed among pneumococcal isolates of ST 124 (serotype 14), while Bloomberg *et al.*[42] observed different AR patterns among isolates of ST 176 (serotype 6B), ST 124 (serotype 14) and ST 156 (serotypes 14 and 19 F). Thus the current study provides evidence of the phenomenon of intraclonal diversity beyond clones and serotypes that have been previously reported. Bloomberg *et al.*[42] pointed out that intraclonal diversity was rare among serotypes of high invasive disease potential, as it was not observed among clones of serotypes 1, 4 and 7 F included in their study. This finding contrasts with the current study, where intraclonal diversity was consistently exhibited by clones (ST 3404 and ST 1233) of serotype 5, a serotype of high invasive disease potential. Nevertheless, it can be observed that while intraclonal diversity occurred among several serotype 5 clones, it did not occur among the more virulent ST 289 (serotype 5) PMEN clone, indicating that intraclonal diversity may be relatively rare among more virulent clones. This is also the case for the virulent ST 618 (serotype 1) clone and also the ST 4012 (serotype 23 F) clone, which is a novel clone and inferred to be virulent, as it was the most frequent cause of mortality (Figure 1). This suggests some association of these virulent clones with stability (uniform genetic content). Dagerhamn *et al.*[47] have demonstrated that some pneumococcal accessory regions may predict genetic relatedness similar to that predicted by MLST, which they attributed to the influence of recombination on variations in housekeeping genes (used for MLST) and as well as accessory regions. Data on intraclonal diversity from the current study further suggests that in some cases accessory regions may also provide better resolution than MLST, as highly genetically similar isolates of the same serotype and MLST can be distinguished by their accessory regions patterns. This shows the potential as a pneumococcal typing scheme based on accessory regions which would provide similar results to MLST but of better resolution. However, it should be noted that typing by analysis of ARs could be especially susceptible to being confounded by horizontal gene transfer.

### Comparative phylogenomics

**Figure 2:**
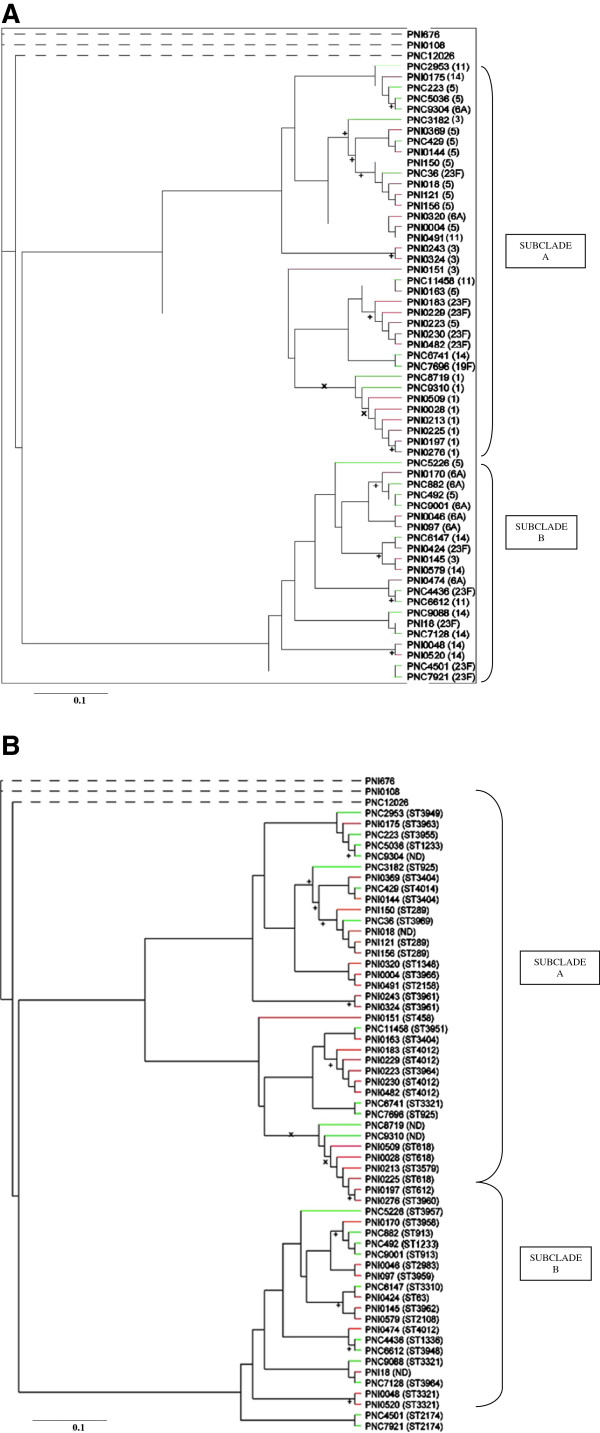
**A: Phylogeny of *S. pneumoniae *isolates (serotype analysis).** Isolate names are shown in brackets while the corresponding serotypes are indicated outside brackets; Non-pneumococcal isolates are shown by dotted lines. Invasive isolates are shown in red while carriage isolates are shown in green; + indicates p = 1.0 while × indicates p > 0.9. **B: Phylogeny of *S. pneumoniae* isolates (sequence type analysis).** Isolate names are shown in brackets while the corresponding MLST are indicated outside brackets; Non-pneumococcal isolates are shown by dotted lines. Invasive isolates are shown in red while carriage isolates are shown in green; STND indicates MLST of the isolate was not determined; + indicates p = 1.0 while × indicates p > 0.9.

The data obtained from microarray analysis was used to generate a phylogenetic tree which is shown in Figure 2. Three of the isolates (PNI676, PNI0108 and PNC12026) subjected to phylogenomic analysis showed a distant association with all the other isolates. These three isolates were subjected to molecular serotyping using another type of microarray [48] to confirm their identity as *S. pneumoniae*. This showed that the three isolates were not *S. pneumoniae* isolates but likely to be a closely related *Streptococcus* species such as *S. mitis* or *S. oralis*, and hence their separation from *S. pneumoniae* isolates in the phylogenetic tree, which confirms the credibility of the phylogenetic relationship among the isolates. MLST of the three isolates also showed that the sequences were divergent from those of known MLST alleles. The three non-pneumococcal isolates were excluded in analysis of the core genome (Section “Core gene set of S. pneumoniae strains”) as well as analysis of putative virulence determinants and accessory regions (Section “Putative virulence determinants and accessory regions”).

Phylogenetic analysis of the *S. pneumoniae* isolates showed two major clades, with each clade comprising a mixture of invasive and carriage isolates of varied serotypes (Figure 2). Despite the heterogeneous clustering of serotypes, all of the eight serotype 1 isolates (six invasive and two carriage isolates) formed a subclade (Figure 2A). Recently, Donati *et al.*[23] constructed a phylogenetic tree based on 44 sequenced pneumococcal genomes covering 19 different serotypes and 24 MLST clonal clusters. By comparison, in this study, the poor correlation observed between a serotype of an isolate and its position in the tree except for serotype 1, agrees well with the study by Donati *et al.*[23]. Similarly, the poor correlation observed between an isolate from an invasive or carriage source and its position in the tree also agrees with the study by Donati *et al.*[23]. The high level of heterogeneity among isolates in the phylogenetic tree of this study is probably due to recombination which occurs frequently among pneumococci. A recent study by Croucher *et al.*[49] found more than 700 recombination events in 240 strains of the PMEN1 (Spain^23F^-1) multidrug-resistant lineage. According to Feil *et al.*[50], evolution of the pneumococcal population is dominated by recombination, and can abolish any deep-rooted phylogenetic signal resulting in a pattern of heterogeneity as observed in this study. The homogeneous clustering observed among the serotype 1 isolates agrees with the uniform distribution of ARs observed among the serotype 1 isolates, and reflects the fact that, because this serotype is rarely carried, it is less likely to undergo recombination. Within the phylogenetic tree, clustering of isolates of the same MLST was observed (Figure 2B), which has also been reported by Donati *et al.*[23] and Dagerhamn *et al.*[47], and provides evidence of the agreement between microarray and MLST. This implies that the frequent pneumococcal recombination did not eliminate phylogenetic signals related to a common ancestor though it may have weakened such signals.

An attempt was made to use MacClade 4 to identify CDSs which were associated with relevant clades and subclades in the *S. pneumoniae* phylogenetic tree (Figure 2). The two major clades formed, were associated with presence/absence of AR6, AR8, AR9 and AR13 (Table 3). These ARs have been reported to have some importance in pneumococcal pathogenicity [21,51,52]. The fact that each clade comprised a mixture of invasive and carriage isolates probably support the earlier claim in this study that ARs may have little relevance in pneumococcal pathogenicity. The formation of the serotype 1 cluster of isolates (Figure 2A) was associated with 10 CDSs, all of which were highly divergent or absent from these isolates.

## Conclusions

The current study is unique in that it is based on a relatively large number (58) of *S. pneumoniae* isolates from the developing world (West Africa), while other studies were based mainly on isolates from developed countries. Comparative phylogenomics of invasive and carriage *S. pneumoniae* isolates identified a number of putative virulence determinants that may be important in the progression of *S. pneumoniae* from the carriage phase to invasive disease. These putative virulence determinants are currently being investigated by mutagenesis to confirm their role in pneumococcal pathogenicity. Virulence determinants that contribute to *S. pneumoniae* pathogenicity are likely to be distributed randomly throughout its genome rather than being clustered in dedicated loci or islands. Compared to other *S. pneumoniae* serotypes, serotype 1 maintains a more uniform genetic content which implies that serotype 1 strains are more likely to be clonally related than strains of other serotypes.

### Limitations

There are a number of limitations of the study. Firstly, the microarray used was based on only two sequenced genomes including TIGR4 and R6 strains, which are reference strains from developed countries rather than the developing world where the study isolates were collected. This means that genes that are absent in the reference strains but present in the study isolates may not be detected. Secondly, it is not known if the genes detected are expressed in vivo or not and if expressed under what conditions. The second limitation is partly addressed by the fact that expressions of some of the virulence genes identified (SP0071, SP0743 and SP1032) have been demonstrated by other investigators [53,54].

## Methods

### Identification of *S. pneumoniae* isolates and extraction of DNA

The study isolates were confirmed to be *S. pneumoniae* by the optochin test [55]. The isolates were purified on 5% blood agar plates and bacterial chromosomal DNA was prepared using the Wizard gDNA purification kit (Promega). The concentration and purity of extracted DNA was determined by means of a NanoDrop® ND-1000 spectrophotometer (NanoDrop, Wilmington, USA).

### Microarray analysis

*S. pneumoniae* genomic DNA extracted from the study isolates and reference strain were analysed using the BμG@S SPv1.1.0 microarray as described previously [43]. This microarray consisted of duplicate spotted PCR products, representing all annotated genes in *S. pneumoniae* strains TIGR4 and R6. Briefly, 1 μg of DNA was labelled by random priming with Klenow polymerase to incorporate either Cy3 or Cy5 dCTP (GE Healthcare) for the reference strain or the test strain, respectively. Equal amounts of the Cy3- and Cy5-labeled samples were copurified through a Qiagen MinElute column (Qiagen), mixed with hybridization solution (4× SSC 0.3% SDS), and denatured at 95°C for 2 min. The labelled sample was loaded on to a prehybridized microarray under one 22 mm by 22 mm Lifter Slip (Erie Scientific), sealed in a humidified hybridization cassette (Corning), and hybridized overnight by immersion in a water bath at 65°C for 16 to 20 h. Slides were washed once in 400 ml 1 × SSC, 0.06% SDS at 65°C for 2 min and twice in 400 ml 0.06 × SSC for 2 min at room temperature. The microarray slides were then scanned with a GMS 418 Scanner (Genetic Microsystems) and spot fluorescence intensities were determined with ImaGene 5.5 (BioDiscovery Inc.). All the *S. pneumoniae* study isolates were hybridized once against the TIGR4 reference strain and the microarray hybridization experiments were repeated for isolates which gave poor hybridization results.

### Microarray data analysis and comparative phylogenomics

Analysis of the microarray data and comparative phylogenomics were carried out with GeneSpring v6.1 (Silicon Genetics). Data were median normalized in GeneSpring and normalized intensity data for each channel from each microarray were used to run GACK (Genomotypying Analysis Charlie Kim), to determine whether genes were present, absent, or divergent [56]. To run GACK analysis, the raw values were divided by the control values for each sample and then transformed into log2 ratio data. This was saved as a tab delimited file and used as the input file for the GACK software. GACK uses the log2 ratio data to categorize CDSs based upon estimated probability of presence (EPP). Computation of EPP was done by dividing the mapped normal curve value (the expected value for a distribution in which all spots have signal present on the hybridized microarray) by the actual observed data distribution value for any given ratio [56]. Two stringent cut-offs were used; ‘present’ is called only if a GACK EPP was ≥100% , ‘absent (or highly divergent)’ was only called in GACK EPP was ≤0% EPP, ‘divergent’ genes were between 0 and 100% EPP. While this cut-off for absent is highly stringent, the stringent hybridisations conditions equate to divergence of greater than approximately 5% which may result in an ‘absent’ call to a coding sequence that is present hence ‘absent/highly divergent’. The resulting assigned CDS from GACK analysis were re-entered into GeneSpring 6.1 and a core genome of the isolates was determined: core genome was defined as the set of genes present in all the isolates investigated. Genetic differences among the isolates were also determined at a significant level of p < 0.05 and Chi square was used to confirm virulence genes (ie genes that were significantly associated with invasive isolates).

The output of GACK was transformed into NEXUS format, and the relationship of the strains was determined based on Bayesian method-based algorithms implemented through Mr Bayes v3.0 software [57]. The resulting phylogenetic trees were viewed using TREEVIEW ( http://taxonomy.zoology.gla.ac.uk/rod/treeview.html). Coding sequences (genes) associated with the phylogenomic relationships of isolates and also the formation of clades and subclades were evaluated using MacClade 4 [58].

### Ethical considerations

The study was approved by the ethics committee of the Medical Research Council (The Gambia). The isolates used were gathered from various laboratories and human subjects were not enrolled in the study.

## Competing interests

The authors declare that they have no competing interests.

## Authors’ contributions

The study was conceived by BWW, RAS, MA and RAA. Microarray experiments were performed by ESD. Bioinformatics analyses were done by RAS, ESD and JH. The manuscript was drafted and revised by ESD, BWW, RAS, MA, RAA and JH.
